# Improving Accuracy in Reverse Total Shoulder Arthroplasty for Acute Proximal Humerus Fractures Using Virtual Surgical Planning: A Comparative Cohort Study

**DOI:** 10.3390/jcm15114150

**Published:** 2026-05-27

**Authors:** Nick Smeitink, Egbert J. D. Veen, Lian Klein Teeselink, Louise H. M. Govaert, Rob F. M. van Doremalen, Gabriëlle J. M. Tuijthof, Femke F. Schröder

**Affiliations:** 1 Department of Orthopaedics, Medisch Spectrum Twente, P.O. Box 50000, 7500 KA Enschede, The Netherlands; 2 Medical 3D Lab, Department of Medical Technology, Medisch Spectrum Twente, P.O. Box 50000, 7500 KA Enschede, The Netherlands; 3 Department of Biomechanical Engineering, University of Twente, P.O. Box 217, 7500 AE Enschede, The Netherlands

**Keywords:** proximal humerus fracture, virtual surgical planning, reverse total shoulder arthroplasty, humeral stem positioning, greater tuberosity healing, range of motion

## Abstract

**Background/Objectives:** Reverse total shoulder arthroplasty (rTSA) is the preferred treatment for elderly patients with complex proximal humerus fractures. Accurate humeral stem positioning remains challenging in these cases due to complex fracture patterns, which may lead to postoperative complications. Virtual surgical planning (VSP) may assist in optimizing humeral stem and greater tuberosity positioning; however, its clinical impact in fracture-related rTSA has not yet been evaluated. This study aimed to assess whether VSP improves postoperative range of motion (ROM). **Methods:** A comparative cohort study was conducted, comprising a prospective VSP group and a retrospective control group. Patients underwent rTSA for proximal humerus fractures. Primary outcomes were ROM during forward elevation, abduction, and external rotation at two months and one year postoperatively. Secondary outcomes included complications, procedure time, greater tuberosity repositioning and healing, and postoperative deviation in humeral stem height compared with the preoperative plan. Statistical analyses included independent *t*-tests, Mann–Whitney U tests, and chi-square tests. **Results:** A total of 48 patients were included: 27 in the VSP group and 21 in the non-VSP group. At two months, abduction was significantly greater in the VSP group (76° vs. 63°, *p* = 0.05). Forward elevation and external rotation were numerically higher in the VSP group but did not reach statistical significance (*p* < 0.1). Stem height deviation was significantly lower in the VSP group (3 mm vs. 12 mm, *p* < 0.001). **Conclusions:** VSP enables more accurate humeral stem positioning in rTSA for proximal humerus fractures. Although no statistically significant improvements in ROM were observed at one year, VSP demonstrated superior accuracy in stem positioning.

## 1. Introduction

Proximal humerus fractures are among the three most common traumatic fractures in the elderly, accounting for approximately 5–6% of all fractures [1]. Most of these fractures are treated non-operatively; however, displaced or comminuted fractures often require surgical intervention to prevent chronic pain and to optimize functional outcomes [2].

Surgical treatment includes open reduction and internal fixation (ORIF) or arthroplasty. ORIF is generally preferred in younger patients, whereas arthroplasty is favored in older patients due to factors such as osteoporotic bone, increased risk of avascular necrosis, malunion and poor function [2]. In elderly patients with complex fractures, reverse total shoulder arthroplasty (rTSA) has become more common than hemiarthroplasty [3]. rTSA alters shoulder biomechanics by shifting the center of rotation medially and inferiorly, thereby reducing reliance on rotator cuff function for range of motion [4], which is often compromised in this population. Despite its advantages, rTSA remains challenging in fracture cases due to complex anatomy and limited intraoperative visibility, which can compromise optimal implant positioning, particularly when relying on conventional landmark-based intraoperative estimation. This may result in complications, such as instability and malposition [5]. In particular, accurate humeral stem positioning is crucial, as excessive stem height may lead to increased deltoid tension and acromial stress, whereas insufficient stem height can result in inadequate soft tissue tensioning and subsequent instability, both of which may impair postoperative range of motion [6,7]. Following proximal humerus fractures, rTSA is associated with a significantly increased risk of revision compared to arthroplasty performed for osteoarthritis (hazard ratio 2.5) [8].

To minimize complications and reduce the need for revision surgery, accurate implant positioning in rTSA is essential [5]. In recent years, computer-assisted techniques such as 3D preoperative virtual surgical planning (VSP) and patient-specific instrumentation have been introduced in orthopedic procedures to improve the accuracy and reproducibility of implant placement. VSP has been proposed to improve the positioning of the reverse shoulder implant as well [9]. This technique involves CT-based fracture segmentation and contralateral mirroring for anatomical reconstruction, followed by virtual planning of the resection plane and humeral stem placement [9]. It aims to optimize both the positioning and height of the humeral stem in relation to the bone and the anatomical repositioning of the greater tuberosity [9]. Tuberosity reconstruction has been associated with improved range of motion and higher patient satisfaction [10,11,12]. It is therefore hypothesized that VSP in rTSA may improve clinical outcomes, reduce complications, and result in more accurate anatomical reconstruction compared with conventional planning methods.

However, the clinical impact of preoperative VSP in rTSA for proximal humerus fractures has not yet been evaluated. The primary aim of this study was to assess whether VSP improves postoperative range of motion in patients undergoing rTSA for proximal humerus fractures. Secondary aims were to evaluate complications, procedure time, greater tuberosity repositioning and healing, and postoperative deviation in stem height.

## 2. Materials and Methods

### 2.1. Study Design

**Figure 1:**
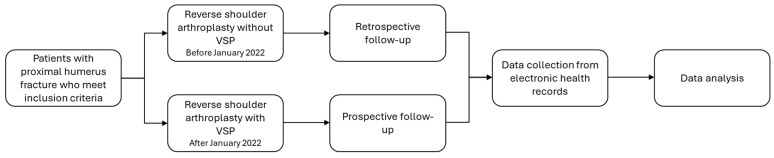
Summary of study design. VSP: virtual surgical planning.

This study was conducted at Medical Spectrum Twente, Enschede, the Netherlands, and used a comparative cohort design, including a prospective intervention group and a retrospective control group with a minimum follow-up duration of one year in both groups. The first group comprised patients with a proximal humerus fracture who underwent rTSA with the aid of VSP. These patients were prospectively recruited and treated from January 2022, following the implementation of virtual planning in clinical practice [9]. The second group comprised patients who underwent the same surgical procedure without preoperative virtual planning, prior to its implementation in our clinic. Patients were included if they had a proximal humerus fracture with an indication for rTSA, including dislocated, comminuted, three- or four-part fractures, treated acutely within 28 days after trauma. Patients with previous surgery or deformity of the affected shoulder or insufficient follow-up were excluded. Their data were retrospectively collected from medical records with a minimum of one year of follow-up after rTSA, covering the period from January 2020 to January 2022. The study design is summarized in Figure 1.

### 2.2. VSP and Surgical Procedure

**Figure 2:**
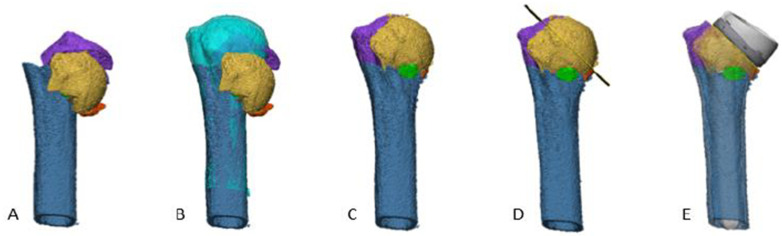
VSP: (**A**) fracture segmentation and visualization, (**B**) mirror and alignment of contralateral humerus, (**C**) humerus reconstruction including tuberosities, (**D**) determination of resection plane, and (**E**) implantation of humeral stem. All fracture parts have different colors for identification.

**Figure 3:**
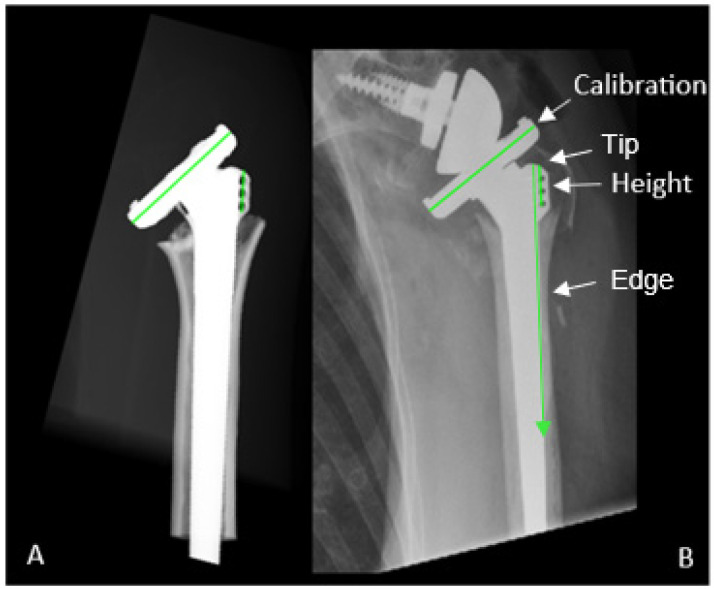
Humeral stem height measurement, calibrated with measurement of humeral tray (see green lines): (**A**) DRR of VSP; (**B**) postoperative radiograph.

**Figure 4:**
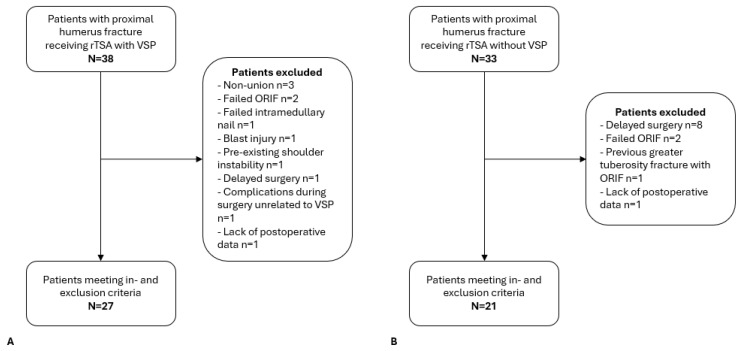
Flowchart of inclusion of (**A**) patients with VSP and (**B**) patients without VSP. ORIF: open reduction and internal fixation. rTSA: reverse total shoulder arthroplasty. VSP: virtual surgical planning. Both groups were comparable in terms of baseline characteristics (Table 1). In both groups, the majority of patients were female, with a mean age of approximately 70 years and a mean BMI of approximately 30 kg/m^2^. Most patients were non-smokers and classified as ASA class II. No statistically significant differences were found between groups regarding gender, age, BMI, smoking status, ASA classification, side of fracture, or medical history of the affected side (Table 1). However, there was a significant difference in the distribution of implanted humeral stem types between the VSP and control groups (*p* < 0.001).

**Figure 5:**
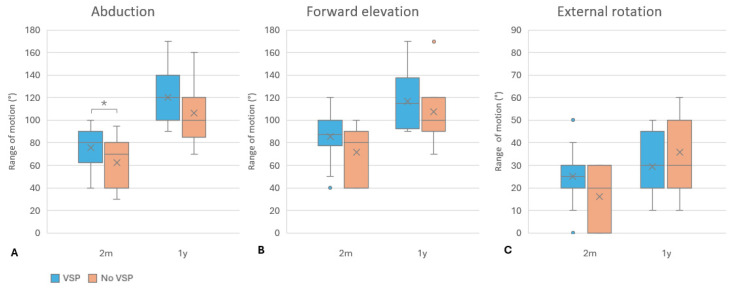
Range of motion (ROM) of (**A**) forward elevation, (**B**) abduction and (**C**) external rotation at 2 months and 1 year for VSP and non-VSP groups. Dots indicate outliers, crosses represent the mean, and an asterisk (*) denotes statistical significance (*p* < 0.05).

The VSP was performed preoperatively using Materialise Mimics and 3-Matic (Materialise, Leuven, Belgium), as previously published by our research group [9]. The workflow consisted of five steps: (1) segmentation of a CT scan of the fractured proximal humerus and, if possible, the contralateral humerus (Figure 2A), (2) mirroring and alignment of the contralateral humerus (Figure 2B), (3) fracture reconstruction, including the tuberosities (Figure 2C), (4) determination of the resection plane (Figure 2D), and (5) implantation and positioning of the humeral stem with visualization of the planned stem height (Figure 2E).

Based on the VSP, the surgical procedure was carried out as follows. rTSA was performed through a deltopectoral approach. First, the glenoid component was implanted using standard surgical techniques as recommended by established professional guidelines and manufacturer instructions, with positioning adapted to individual patient anatomy. Hereafter, the fractured proximal humerus was addressed. During humeral stem implantation, the operating orthopedic surgeon reviewed the VSP preoperatively and used it as an intraoperative reference, which was displayed on the operating room monitor. The humeral stem was positioned to replicate the preoperative plan as closely as possible using sterile measuring tape for manual verification of stem height. Finally, the greater tuberosity was reattached whenever feasible, based on intraoperative assessment of fracture configuration and soft tissue condition. All procedures in both the VSP and control groups were performed by the experienced shoulder surgeons. Three types of humeral stems from Zimmer Biomet (Warsaw, IN, USA) were used: the Comprehensive Fracture Stem, the Anatomical Shoulder Fracture Stem, and the Anatomical Domelock Stem.

### 2.3. Data Collection and Measurements

General information and outcome measures were collected from the electronic health record. Patient characteristics included gender, age, BMI, American Society of Anesthesiologists (ASA) physical status classification, smoking status, side of fracture, and any prior surgery or relevant history of the affected extremity. Surgical details comprised procedure time, achievement of greater tuberosity repositioning (as assessed on postoperative radiographs by an orthopedic surgeon), intraoperative complications, and postoperative radiographs and reports. Physical examination results at two months and one year included a range of motion during forward elevation, abduction, and external rotation [13]. Postoperative complications within one year were also recorded.

To assess the accuracy of humeral stem placement, the stem height of the humeral stem relative to the humeral shaft on postoperative radiographs was compared with digitally reconstructed radiographs (DRRs) (Figure 3). For the group with VSP, DRRs were generated from the preoperative VSP in Materialise Mimics. For patients without preoperative VSP, a VSP was retrospectively created and DRRs were generated for stem height comparison in both groups. The DRRs were adjusted until the DRR resembled the postoperative anterior–posterior radiograph (Figure 3). The stem height (in mm) was measured from the upper tip of the humeral stem to the highest lateral spike of the humeral shaft, along the edge of the humeral stem, using the known humeral tray width for calibration (Figure 3). Measurements were performed using MATLAB (R2023a, MathWorks, Natick, MA, USA). Each measurement was performed by a single observer and repeated three times to reduce measurement variability, and the mean value was used for analysis. Finally, the difference in stem height between the postoperative radiograph and the DRRs was calculated.

### 2.4. Statistical Analysis and Sample Size Calculation

A priori power analysis determined that a minimum of 30 patients per group was required to detect a statistically significant difference in abduction of 15 degrees or more, which has been shown to be clinically relevant [14]. This estimated effect size was based on preliminary data and conducted with a significance level of *α* = 0.05 and a power of 80% (1 − *β* = 0.80), resulting in a total sample size of 60 patients, with 30 allocated to each group. Abduction was selected as the primary parameter for sample size calculation due to its representative value for overall shoulder function and clinical relevance following rTSA.

Patient characteristics, surgery details, imaging and physical examination results, and complications were presented using descriptive statistics. The normality of the distribution of continuous variables, including BMI, procedure time and range of motion, was evaluated by visually inspecting histograms and performing Shapiro–Wilk tests. Normally distributed continuous variables were presented as means with standard deviation (SD), while non-normally distributed variables were summarized using median and interquartile range (IQR). Continuous variables were compared between both groups using either independent-sample *t*-tests for normally distributed data or Mann–Whitney U tests for non-normally distributed data. Dichotomous and categorical variables were summarized using frequencies (n) and percentages (%). Chi-square tests or Fisher’s exact tests with a possible Freeman–Halton extension were used to compare these dichotomous and categorical variables, among which were smoking status, ASA, and occurrence of complications. Data distributions were visualized using boxplots where relevant. To maximize the number of included observations, missing data were handled through available-case analysis, whereby each analysis included all cases with complete data for the relevant variables. No multivariable analyses were performed due to the limited sample size. As such, potential confounding was not adjusted for.

For all analyses, *p*-values of 0.05 and lower were considered significant. Analyses were performed using SPSS version 29 (IBM, New York, NY, USA).

## 3. Results

### 3.1. Patient Characteristics

A total of 71 patients with a proximal humerus fracture underwent rTSA between January 2020 and July 2024 (Figure 4). Of these, 38 patients were treated with VSP between January 2022 and July 2024, and 33 patients received rTSA without VSP between January 2020 and January 2022. Nine patients in the VSP group and twelve in the non-VSP group were excluded for various reasons, including delayed surgery (>28 days) and failed ORIF or conservative treatment (Figure 4). This resulted in 27 patients in the VSP group and 21 patients in the non-VSP group, who were included in the final analysis.

**Table 1:** Patient characteristics stratified by VSP.

Variable	No VSP (N = 21)	VSP (N = 27)	*p*-Value
Gender, *n* (%)			0.4 ☨
Male	1 (5)	4 (15)	
Female	20 (95)	23 (85)	
Mean age, years (SD)	70 (8)	72 (7)	0.4 ‡
Mean BMI, kg/m^2^ (SD)	28 (5)	29 (5)	0.8 ‡
Smoking, *n* (%)			0.6 ☨
Yes	3 (14)	2 (7)	
No	18 (86)	25 (93)	
ASA classification, *n* (%)			0.6 ☨
I	1 (5)	0 (0)	
II	14 (67)	18 (67)	
III	6 (28)	9 (33)	
Side of fracture, *n* (%)			0.9 †
Right	11 (52)	13 (48)	
Left	10 (48)	14 (52)	
Medical history on affected side, *n* (%)			0.7 ☨
Yes	5 (24)	7 (26)	
No	16 (76)	20 (74)	
Implanted humeral stem, *n* (%)			
Comprehensive Fracture Stem	4 (19)	18 (67)	<0.001 ☨
Anatomical Shoulder Fracture Stem	8 (38)	9 (33)	
Anatomical Domelock Stem	9 (43)	0 (0)	

† Chi-square test. ☨ Fisher (–Freeman–Halton) exact test. ‡ Independent-sample *t*-tests. ASA: American Society of Anesthesiologists. BMI: body mass index. SD: standard deviation. VSP: virtual surgical planning.

### 3.2. Primary Outcome Measures

An overview of ROM values after two months and one year is shown in Figure 5. After two months, a statistically significant difference was found in abduction, with the VSP group showing a higher mean value (76°, SD 19°) compared to the non-VSP group (63°, SD 23°; *p* = 0.05) (Figure 5A). Forward elevation showed a higher median in the VSP group (88°, IQR 78–100°) than in the non-VSP group (80°, IQR 40–90°), although this difference was not statistically significant (*p* = 0.1) (Figure 5B). External rotation also showed a slightly higher median in the VSP group (25°, IQR 20–30°) compared with the non-VSP group (20°, IQR 0–30°), with a *p*-value of 0.06 (Figure 5C).

At one year, ROM improved for all movements in both groups. Abduction remained higher in the VSP group (120°, SD 21°) compared to the non-VSP group (106°, SD 27°), although the difference was not statistically significant (*p* = 0.09) (Figure 5A). Forward elevation reached a mean of 117° (SD 23°) in the VSP group and 108° (SD 30°) in the non-VSP group (*p* = 0.4). External rotation showed similar median values in both groups (30°), with overlapping interquartile ranges and no significant difference (*p* = 0.2). Overall, no statistically significant differences in ROM were observed between groups at one year.

### 3.3. Secondary Outcome Measures

**Figure 6:**
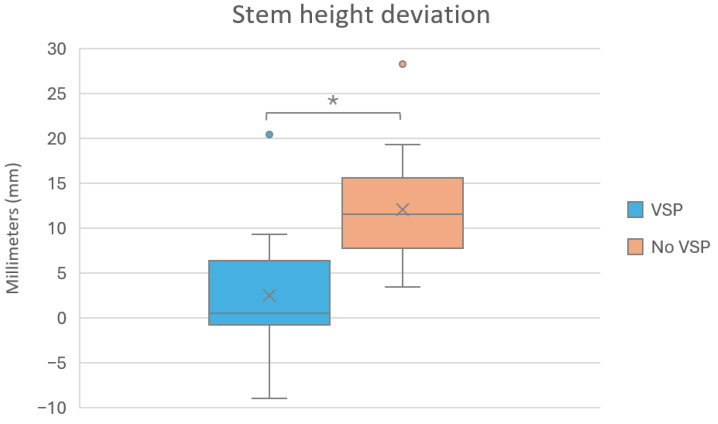
Boxplot of stem height difference between postoperative radiographs and DRRs for VSP and non-VSP groups. Dots indicate outliers, crosses represent the mean, and an asterisk (*) denotes statistical significance (*p* < 0.05).

Of the secondary outcome measures, only stem height deviation differed significantly between the groups (*p* < 0.001). Deviations in stem height occurred in both directions, with stems being positioned either more superior or more inferior than planned. The mean difference in stem height between the DRRs and the postoperative radiographs was 3 mm (SD 6 mm) in the VSP group, compared to 12 mm (SD 6 mm) in the non-VSP group (Figure 6).

No intraoperative or early complications occurred in either group. Within one year, two complications were observed in the VSP group (7%). One patient developed periarthritis with heterotopic ossification on postoperative radiographs. The other patient experienced tingling in the hand after surgery, which resolved spontaneously. Three patients in the non-VSP group experienced complications (14%). All complications were radiographic findings reported by the radiologist on postoperative radiographs: two cases of periprosthetic lucency and one case of heterotopic ossification. The overall complication rate did not differ significantly between the groups (*p* = 0.4).

**Figure 7:**
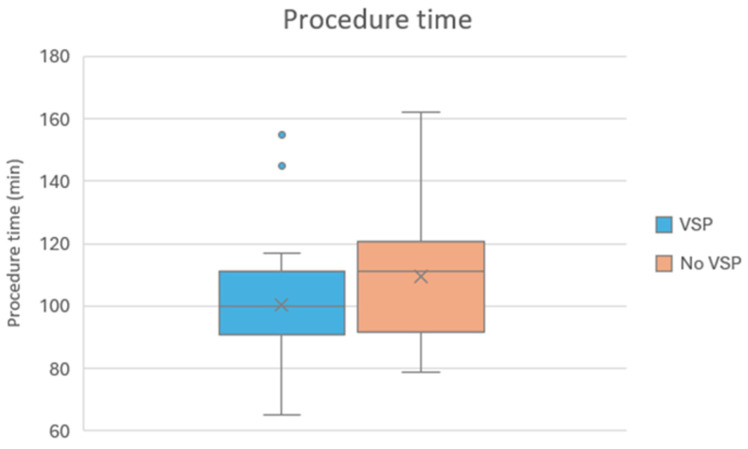
Boxplot of procedure time comparing VSP and non-VSP groups. Dots indicate outliers, and crosses represent the mean.

The procedure time for the VSP group and the non-VSP group is displayed in Figure 7. The mean procedure duration was nine minutes shorter in the VSP group (VSP: 101 min, no VSP: 110 min, SD 20). This difference was not statistically significant (*p* = 0.1).

In the VSP group, the greater tuberosity was successfully repositioned in 23 patients (85%). Of those 23, thirteen (48%) healed anatomically, eight (30%) non-anatomically, and two (7%) were resorbed within one year. In sixteen patients in the non-VSP group (76%), the greater tuberosity was reattached. Nine (43%) healed anatomically, six (29%) non-anatomically, and one (5%) was resorbed during the follow-up period. Although anatomical healing of the greater tuberosity appeared slightly more frequently in the VSP group, the difference was not statistically significant (*p* = 0.9), indicating comparable healing outcomes between the groups.

## 4. Discussion

The aim of this study was to evaluate whether VSP improves postoperative ROM in patients undergoing rTSA for proximal humerus fractures. Although statistically significant improvements in ROM were not observed in one year, the ROM of abduction was significantly greater in the VSP group after two months, which may suggest accelerated early postoperative recovery. The differences in ROM at one year were not statistically significant, yet they may still hold clinical relevance in larger sample sizes. Even small improvements in shoulder mobility can impact patients’ ability to perform daily tasks independently [15]. Among the secondary outcomes, only stem height relative to the humeral shaft was found to be statistically significant between patients with and without preoperative VSP, suggesting that VSP may contribute to more accurate and consistent implant positioning. No significant differences were observed in complication rates, tuberosity repositioning and healing, or procedure time between the two groups. Moreover, no dislocations were observed, which is a potential complication associated with deviated stem positioning. As the study was not powered to detect differences in these secondary outcomes, these results should be interpreted with caution. Nevertheless, the observed difference in procedure time may be of clinical relevance as well, especially in high-volume surgical settings, as this would allow more efficient use of operating room resources and potentially increase surgical throughput without compromising patient safety.

Compared to previous studies reporting range of motion after rTSA for proximal humerus fractures, with forward elevation of 122–129°, abduction of 95–110°, and external rotation of 20–46° after two years [16,17,18,19], the present study showed comparable outcomes at one year for patients with and without VSP. This indicates similar but not superior results. It should be noted that the follow-up of this study was limited to one year. While no prior studies have evaluated VSP specifically for rTSA in fracture cases, VSP, possibly combined with a patient-specific guide (PSG), has demonstrated improved surgical accuracy, implant positioning, and procedural efficiency in other orthopedic procedures, such as ORIF for proximal humerus fractures [20], sacroiliac joint fusion [21], corrective osteotomies [22,23], and orthopedic trauma [24,25]. These findings suggest that VSP may offer similar benefits when applied to rTSA, particularly in achieving the preoperatively planned stem height. Although PSGs were not used in the current study, their integration could be a valuable addition in future applications. Finally, complication rates and greater tuberosity healing were also comparable with those reported in the literature [16,17,26].

While the findings provide valuable insights into the use of VSP in rTSA, strengths and limitations should be considered. The main strength of this study is its innovative character, focusing on the integration of advanced surgical planning technology in the treatment of complex proximal humerus fractures. By evaluating VSP in a clinical setting, the study directly addressed its potential clinical relevance. Additionally, VSP enhances the translation of the preoperative plan into the operative field by providing detailed 3D visualization, thereby supporting the surgeon’s mental reconstruction of patient-specific anatomy. Moreover, the inclusion of clinical, surgical, and radiological outcomes allows for a comprehensive assessment of the impact of VSP on both surgical procedure and patient recovery and functionality.

The study also has limitations. First, the study design was not randomized, which introduces a risk of selection bias and limits the ability to draw causal inferences. Although a randomized controlled design would have been preferable, this was not feasible after the implementation of VSP in clinical practice, as surgeons considered it unethical to withhold a potentially beneficial planning tool. Consequently, patients were no longer allocated to treatment groups at random, and systematic differences between groups may have arisen based on the timing of surgery or patient characteristics rather than the intervention itself. Second, the sample size did not meet the target set by the power analysis, thereby limiting the statistical power and the ability to adjust for potential confounding factors. Furthermore, the study was powered for the primary outcome (abduction), but not for secondary outcomes, which increases the risk of type II error for these analyses. Additionally, missing data, including PROMs, particularly in the retrospective group, limited the ability to assess subjective patient recovery and patient satisfaction. However, it has been questioned whether PROMs reliably correlate with patient satisfaction following rTSA [27]. Furthermore, different humeral stems were used during the study period, with a significantly different distribution between groups. This may represent a potential source of confounding. However, previous studies have shown no significant differences in clinical outcomes between fracture-specific and conventional stems in rTSA for proximal humerus fractures [8,28]. Therefore, we expect the impact of this difference on our primary outcomes to be limited. In addition, the assessment of stem height deviation was performed by a single observer and based on visual matching of DRRs, which is not a validated method and may introduce some measurement and observer bias. Nonetheless, the method was applied consistently across cases and offered a pragmatic way to compare planned versus achieved stem height. In the retrospective group, the VSP used for stem height analysis was generated from lower-quality historical CT scans without contralateral reference data, which may have slightly influenced the accuracy of preoperative planning. However, these differences are unlikely to have substantially affected the comparative outcomes.

Despite these limitations, the findings provide meaningful insights into the potential clinical role of VSP in rTSA for proximal humerus fractures. The observed possible early functional advantage and the improved accuracy in stem height positioning suggest that it may enhance the accuracy and consistency of the surgical procedure. These aspects could contribute to improved long-term outcomes, although this remains speculative and requires longer follow-up, including implant survival analysis. The trend toward shorter procedure times and the positive acceptance of VSP among surgeons further demonstrate its feasibility and potential value in routine practice.

As the first clinical comparative cohort study evaluating VSP in rTSA for proximal humerus fractures, the results are promising and highlight that statistical significance does not always reflect clinical relevance. Future studies should aim to confirm these findings in larger, prospective, and preferably randomized multicenter cohorts. The inclusion of PROMs and cost-effectiveness analyses will be essential to fully understand the broader impact of VSP on patient satisfaction, clinical workflow, and healthcare resource allocation.

## 5. Conclusions

This is the first clinical cohort study investigating the use of VSP in rTSA for proximal humerus fractures. VSP appears to enable more accurate humeral stem positioning. Although improvements in ROM were observed, they did not reach statistical significance after one year given the current small sample size, but they may still hold clinical relevance in larger sample sizes. These exploratory findings demonstrate the feasibility and potential value of VSP in clinical practice, particularly in complex fracture cases, but require confirmation in larger, randomized, prospective studies.

## Data Availability

The data presented in this study are available upon request from the corresponding author due to ethical and privacy restrictions.

## Abbreviations

The following abbreviations are used in this manuscript:

ASA	American Society of Anesthesiologists
BMI	Body Mass Index
CT	Computed Tomography
DRR	Digitally Reconstructed Radiograph
IQR	Interquartile Range
ORIF	Open Reduction and Internal Fixation
PROM	Patient-Reported Outcome Measure
PSG	Patient-Specific Guide
ROM	Range of Motion
rTSA	Reverse Total Shoulder Arthroplasty
SD	Standard Deviation
VSP	Virtual Surgical Planning
